# Career calling and safety behavior among nurses: a cross-sectional study based on latent profile analysis

**DOI:** 10.3389/fpsyg.2024.1503051

**Published:** 2025-01-07

**Authors:** Linli Xie, Sijia Xie, Yan Yu, Jie Jing, Min Shi, Lingli Dai

**Affiliations:** ^1^Department of Neurology Intensive Care Unit, Sichuan Provincial People’s Hospital, University of Electronic Science and Technology of China, Chengdu, China; ^2^Department of Nursing, Sichuan Provincial People’s Hospital, University of Electronic Science and Technology of China, Chengdu, China; ^3^Department of Day Surgery Unit, Sichuan Provincial People’s Hospital, University of Electronic Science and Technology of China, Chengdu, China; ^4^Department of Neurology, The Second People’s Hospital of Yibin, Yibin, Sichuan, China; ^5^Department of Gastroenterology, The First People’s Hospital of Yunnan Province, The Affiliated Hospital of Kunming University of Science and Technology, Kunming, Yunnan, China

**Keywords:** nurses, latent profile analysis, career calling, safety behavior, China

## Abstract

**Objective:**

This study aimed to clarify the subgroups of career calling among Chinese nurses, explore the factors correlated with the subgroups, and investigate the relationship between nurse safety behavior and different profiles of career calling.

**Methods:**

A cross-sectional study of 2,567 nurses from 25 hospitals in China was conducted from February to September 2023. A latent profile model of nurses’ career calling was analyzed using Mplus 7.4. The influencing factors of each profile were analyzed by multinomial logistic regression analysis. The hierarchical regression analysis was used to examine the relationship between nurse safety behavior and different profiles of career calling. The STROBE guidelines were followed in this research.

**Results:**

Three distinct latent profiles were identified: “low-calling” type (12.4%), “medium-calling” type (54.4%), and “high-calling” type (33.2%) groups, respectively. Gender and number of night shifts per month were identified as factors influencing the latent profiles of nurses’ career calling. The different categories of career calling significantly predicted the nurse safety behavior (Δ*R*^2^ = 0.307, *p* < 0.001).

**Conclusion:**

This study suggests that nurses experience different types of career calling. The different categories of career calling are significantly associated with the nurse safety behavior. Consequently, administrators should pay attention to the differences in individual career calling and develop targeted intervention strategies to facilitate nurses’ career calling based on the influences of the different underlying profiles and develop enhancement strategies to strengthen nurses’ safety behaviors to ensure patient safety.

## 1 Introduction

Career calling has been one of the most discussed topics in the occupational psychology field (Wu et al., 2024). Career calling refers to an individual’s innate passion and drive for their work, believing that their profession allows them to realize self-worth and life’s purpose (Duffy et al., 2011). In the healthcare industry, career calling is a developmental construct that is particularly important for nurses to make career decisions and set career goals (Praskova et al., 2014). As the nursing shortage worsens, the role of career calling in stabilizing the nursing workforce has increasingly become the focus for global scholars. Previous studies confirmed that embracing a sense of calling can strengthen nurses’ career commitment and reduce burnout (Xie et al., 2024). A considerable amount of literature has confirmed that career calling not only promotes nurses’ work engagement (Wu and Lee, 2020), but also facilitates nurses’ career development (Dik et al., 2019), improves their job satisfaction (Tai et al., 2022), and reduces burnout (Jin et al., 2023). By reducing nurses’ turnover intention (Hong et al., 2023; Li et al., 2024), career calling can stabilize the nursing workforce and contribute to the sustained development of nursing quality care.

Career calling as personal resources can serve as an important positive factor in enhancing the quality of care (Jin et al., 2023; Kim et al., 2023). However, it is worth noting that nurses’ feelings about career calling are not static. Studies show that career calling is an evolving process rather than a one-time event (Vianello et al., 2019). This dramatic change in career calling may pose a threat to the quality of nursing care. There are many open questions about how career calling develops in individuals, how it changes through time, and what predicts these changes are still unknown. Therefore, it is necessary to explore in depth the risk factors that affect nurses’ career calling and seek effective measures to improve nurses’ career calling to ensure that they can better deliver care services for patients.

Patient safety is a fundamental principle of health care and is now being recognized as a large and growing global public health challenge (World Health Organization, 2024). Many countries have developed patient safety action policies and strategies, action plans and programs for the improvement of patient safety. Previous studies have shown that nurse safety behavior is closely related to patient safety and the quality of care (Seo and Lee, 2022; Seo and Lee, 2023). Yet how to improve nurses’ safety behaviors in the healthcare workplace has always been a pressing concern. Both individual and systemic factors can influence nurse safety behavior (Vaismoradi et al., 2020). Among them, career calling is confirmed as a key individual factor that impacts the nurses’ adherence to patient safety principles and safety behaviors (Lee and Hwang, 2024). However, little is known about whether nurses have different career calling profiles and the link between career calling and nurse safety behavior is still unclear. Therefore, exploring the relationship between nurse safety behavior and nurses’ different career calling profiles will enable the implementation of targeted interventions to provide personalized assistance to nurses and strengthen the quality of care.

Latent profile analysis (LPA) enables the examination of how variables interact and influence outcomes within and between individuals (Gabriel et al., 2015). This approach can gain insight into the effects of career calling on nurses’ safety behaviors, identifying the group to which each nurse belongs. In this way, it is possible to guide appropriate interventions to address the unique needs of each group and provide appropriate guidelines for improving safety behaviors among nurses (Zhang et al., 2023). Therefore, this study aimed to reveal the subgroups of the career calling constructs among nurses using an individual-centered approach and to explore the relationship between different profiles of career calling and nurses’ safety behaviors. The results of the study can be applied to develop and test interventions to provide individualized interventions to enhance nurses’ career calling level and safety behaviors.

## 2 Theoretical background and hypothesis

Self-determination theory (SDT) (Ryan and Deci, 2000) is a comprehensive framework for understanding human motivation. SDT suggests that an individual’s motivation can be categorized into intrinsic and extrinsic motivation, both of which play a vital role in driving an individual’s behavior. The theory uniquely emphasizes autonomy, competence and relatedness as fundamental human needs (Ryan and Deci, 2000). It posits that when these needs are fulfilled, employees are motivated by autonomy, which leads to positive psychological perceptions and favorable behaviors toward the organization.

The sub-theories of SDT include Causality Orientation Theory (COT) (Reeve, 2023), which explains how individuals differ in their approaches to engaging with their environments and managing their behaviors. COT identifies and evaluates three distinct causality orientations: the autonomy orientation, where individuals act based on their genuine interest and appreciation for what is happening; the control orientation, which emphasizes rewards, achievements, and external approval; and the impersonal or amotivated orientation, marked by feelings of anxiety related to one’s competence.

Based on the self-determination theory, internal drive and emotions are the sources of self-determination. Therefore, career calling is a strong motivation for nursing work. However, the research on the antecedents of career calling is relatively scarce, and its mechanisms are still unclear. A recent study has shown that motivation is a complex phenomenon that is influenced by a variety of factors, such as biology, psychology, social and cultural factors, economics, the environment, and external stressors (Bandhu et al., 2024). Guided by the Causality Orientation Theory, individuals exhibit personal differences in their intrinsic motivation. Consequently, it is reasonable to assume that there are variations in nurses’ career calling due to individual differences in motivational orientation within today’s rapidly evolving and high-pressure nursing environment.

Therefore, we made the following hypothesis:

*H1*: Different categories of career calling exist among nurses;

*H2*: There are differences between socio-demographic characteristics of nurses and latent profiles’ characteristics of career calling.

In the area of healthcare research, increasing evidence suggests that individual motivation positively influences work behaviors among nurses. Intrinsic motivation at work will foster nurses’ work engagement and prevent them from burning out, particularly when job demands are high (Kohnen et al., 2023). According to the self-determination theory, career calling can autonomously mobilize intrinsic motivation to be motivated and passionate at work, and achieve higher levels of work performance. Previous research indicated that career calling can positively impact nurses’ positive behaviors that benefit the organization (Afsar et al., 2018).

Nurse safety behavior refers to the behavioral state of the nurse when performing nursing tasks for patients, which includes compliance with the nursing code of conduct and core system, and implementation of safety nursing measures (Ma et al., 2023). Based on SDT theory, nurses are more inspired to work autonomously in patient care due to the strong internal motivation stemming from career calling. A strong sense of career calling serves as a significant motivator, making it easier for nurses to wholeheartedly embrace their roles. Nurses with higher career calling are more likely to activate and anticipate the planning of safety nursing practice, enhance their adherence to patient safety principles, and learn about safety nursing knowledge to improve safety care capacity. In addition, nurses with higher career calling will also more actively pursue collaboration with the health care work team to achieve the work goals, thus they may perform better safety behaviors.

Therefore, we proposed the following hypothesis:

*H3*: The profiles of career calling were positively associated with nurse safety behavior.

Currently, the research focuses on variables of career calling is scarce and fragmented, and there is still a lot of controversy in the academic community as to whether intrinsic or extrinsic factors are dominant in the generation of nurses’ career calling. This research aimed to systematically analyze the key factors affecting nurses’ career calling from multiple dimensions to provide a new perspective for constructing an integrative influence model of nurses’ career calling. In addition, by using the latent profile analysis, we tried to identify the hidden subgroup structure and reveal the characteristics of each potential subgroup of nurses’ career calling. In summary, examining the three hypotheses will increase the understanding of the career calling among nurses and provide more targeted guidance for developing interventions to facilitate the career calling of nurses and the quality of care.

## 3 Materials and methods

### 3.1 Design, setting, and participants

A descriptive, cross-sectional study following the STROBE checklist was adopted. A total of 2,567 nurses from 25 hospitals in China were surveyed. Nurses in this study gave informed consent and participated in the study voluntarily. Criteria for eligible nurses were as follows: (1) holding a valid Chinese nursing license; (2) registered nurses working in clinical for at least 1 year; (3) willingness to participate in this study. Nurses on temporary leave, nursing students, or those who had experienced serious health issues within the past 2 years were excluded.

### 3.2 Data collection

Data collection took place from February to September 2023. Questionnaire Star software^1^ was used to conduct the online survey. With the consent of the hospital authorities, the researcher forwarded the questionnaire to the nurses via WeChat, and the questionnaire was completed anonymously and voluntarily after obtaining informed consent. To improve the quality of the study, two items were used to assess whether participants were attentive to their answers (“I have never lied about anything.” and “I never hide my mistakes.”). Successful submission is achieved only after the questionnaire has been filled out completely and each IP address can only be filled in once. At the end of the survey, the collected questionnaires were checked by two researchers, questionnaires that did not pass the test items, took less than 3 min to complete the full questionnaire, or selected the same number in more than two scales were excluded. A total of 3,000 questionnaires were distributed in this study, and 2,567 were validly recovered, with an effective recovery rate of 85.57%.

### 3.3 Measures

#### 3.3.1 Questionnaire on demographic and work-related characteristics

A researcher-designed questionnaire was used, which included gender, age, education level, recruitment methods, years of work experience, number of night shifts per month, and professional title.

#### 3.3.2 The nurse safety behavior questionnaire (NSBQ)

The Nurse Safety Behavior Questionnaire (NSBQ) (Shih et al., 2008) was used to assess nurses’ safety behaviors, which was a single-dimension scale with 12 items (e.g., “I strive to maximize patient safety in my job”). The items were rated on a 5-point Likert scale ranging from 1 (strongly disagree) to 5 (strongly agree). The higher the score, the better nurses’ performance in safety behaviors. The Cronbach’s alpha for this scale in this study was 0.944.

#### 3.3.3 The career calling questionnaire of 12 (CQ12)

The Career Calling Questionnaire of 12 (CQ12) (Dobrow and Tosti-Kharas, 2011) was used in this study, which was translated into Chinese version by Fei (2015). The scale is unidimensional, includes 12 items, and is scored using a Likert 5-point scale ranging from 1 (strongly disagree) to 5 (strongly agree). (e.g., “When I describe myself to others, the first thing that comes to mind is that I am a nurse”). The total score on the scale ranges from 5 to 60, with higher scores indicating higher levels of career calling among nurses. The Cronbach’s alpha for this scale in this study was 0.937.

### 3.4 Ethics statement

The protocol was approved by the Ethics Committee of Sichuan Academy of Medical Science & Sichuan Provincial People’s Hospital (No.2023–03, approved 7/January/2023). Approval was obtained from the management supervisor of each sample hospital before data collection. The data collected from the participants were anonymous and completely confidential. The Participants were given informed consent and participated in the study voluntarily, and they had the right to withdraw at any time.

### 3.5 Data analysis

The Latent Profile Analysis (LPA) was used to identify subgroups of individuals who share a similar profile of scores on career calling. The mean variables of career calling were measured to assess the trait indicator variables. The Mplus 7.4 software was used in the LPA process. The Akaike Information Criterion (AIC), Bayesian Information Criterion (BIC), sample size-adjusted BIC, and an entropy test were used to select the model. The Bootstrap Likelihood Ratio Test (BLRT) and Lomond-Dale-Rubin corrected likelihood ratio (LMR) were used to compare the difference in fit between the models, with information entropy values closer to 1 representing more accurate categorization (Chen et al., 2023). A stepwise approach was used to determine the number of latent profiles that best characterize the data and sample. Multinomial logistic regression was used to examine the relationship between gender, age, professional title, years of work experience, number of night shifts per month, and recruitment methods with profile class membership. Hierarchical regression analysis was used to examine the association between profile class memberships and nurse safety behavior while incorporating sociodemographic characteristics as covariates into the model. All variables with univariate *p*-values<0.05 were chosen as independent variables for the multinomial regression models.

## 4 Results

### 4.1 Demography and work-related characteristics

A total of 3,000 nurses participated in this study, 433 nurses were excluded due to the invalid response or less than 3 min of answer time. Two thousand five hundred and sixty-seven questionnaires were included for data analysis eventually. There were 2,518 women (98.1%). The average age of nurses was 33.69 years (SD = 6.98), ranging from 21 to 59 years of age. There were 1792 nurses with a bachelor’s degree or above (69.8%). The majority were senior nurses and supervisor nurses (82.4%). Most of the nurses had more than 5 years of working experience (85.9%). Additionally, most of the participants were contract staff (75.3%).

### 4.2 The profiles of career calling

The potential profile analysis of the career calling scores of 2,567 nurses was conducted, and 5 models were established. The fitting indexes of each model are shown in Table 1. Class 3 had the highest entropy value of 0.963, the decrease of BIC value from 3 to 4 categories became slower. According to the simplicity and interpretability of the model, the 3-category model was selected as the optimal potential profile model for nurses’ career calling in this study.

**Table 1:** Nurse career calling latent profile model fitting indicators (*n* = 2,567).

Model	AIC	BIC	aBIC	Entropy	LMR (*p*-value)	BLRTR(*p*-value)	Probability
1-profile	87453.97	87594.37	87518.12	–	–	–	–
2-profile	73180.07	73396.53	73278.98	0.957	<0.01	<0.01	0.256/0.744
3-profile	64856.09	65148.62	64989.75	0.963	<0.01	<0.01	0.124/0.544/0.332
4-profile	60980.68	61349.26	61149.09	0.957	<0.01	<0.01	0.188/0.039/0.332/0.191
5-profile	59654.29	60098.93	59857.46	0.952	<0.01	<0.01	0.044/0.066/0.410/0.188/0.292

AIC, Akaike information criterion; BIC, Bayesian information criteria; aBIC, adjusted Bayesian information criteria; LMR, Lo–Mendell–Rubin test; BLRT, bootstrap likelihood ratio test.

### 4.3 Characterization of potential profiles of nurses’ career calling

**Figure 1:**
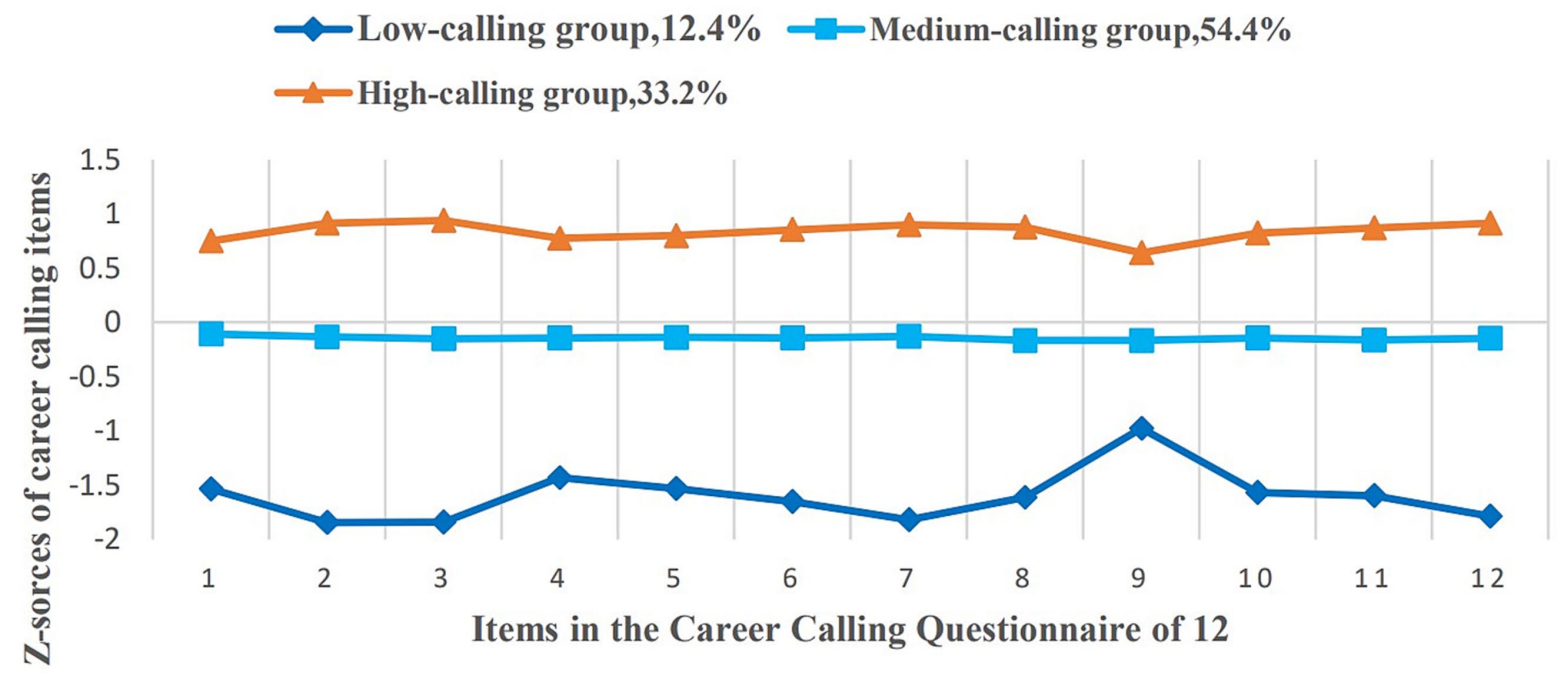
Latent profiles of career calling among nurses.

The results of the potential profiling showed that category 1 had 318 cases (12.4%), category 2 had 1,397 cases (54.4%), and category 3 had 852 cases of nurses (33.2%). The characteristics of the 3 potential categories of nurses’ career calling are shown in Figure 1. Based on the fluctuation of the mean value of each entry, profile 1 was named the “low-calling”, profile 2 was named the “medium-calling”, and profile 3 was named the “high-calling”.

### 4.4 Demographics and characteristics by latent profile

The results showed that there were significant differences among the three profiles in terms of gender, age, education level, professional title, and years of work experience (*p* < 0.001), the results are shown in Table 2.

**Table 2:** Demographic and characteristics by latent profile (*N* = 2,567).

Variables	Profile 1 (*n* = 318)	Profile 2 (*n* = 1,397)	Profile 3 (*n* = 852)	Chi-square	*p*
	*n* (%)	*n* (%)	*n* (%)		
Gender					
Male	13 (4.1%)	22 (1.6%)	14 (1.6%)	9.219	0.01
Female	305 (95.9%)	1,375 (98.4%)	838 (98.4%)		
Age					
20 ~ 30	122 (38.4%)	406 (29.1%)	172 (20.2%)	86.552	<0.001
30 ~ 40	166 (52.2%)	796 (57.0%)	463 (54.3%)		
>40	30 (9.4%)	195 (14.0%)	217 (25.5%)		
Education level					
Junior college	88 (27.7%)	430 (30.8%)	258 (30.3%)	1.187	0.552
Bachelor’s and above	230 (72.3%)	967 (69.2%)	594 (69.7%)		
Professional title					
Nurse	53 (16.7%)	171 (12.2%)	105 (12.3%)	46.387	<0.001
Senior nurse	140 (40.0%)	547 (39.2%)	283 (33.2%)		
Supervisor nurse	118 (37.1%)	632 (45.2%)	396 (46.5%)		
Deputy/director nurse	7 (2.2%)	47 (3.4%)	68 (8.0%)		
Years of experience					
<5	63 (19.8%)	197 (14.1%)	103 (12.1%)	69.92	<0.001
5 ~ 10	102 (32.1%)	403 (28.8%)	174 (20.4%)		
10 ~ 15	103 (32.4%)	490 (35.1%)	289 (33.9%)		
≥15	50 (15.7%)	307 (22.0%)	286 (33.6%)		
Number of night shifts per month					
None	43 (13.5%)	325 (23.3%)	252 (29.6%)	45.312	<0.001
1 ~ 5	72 (22.6%)	339 (24.3%)	213 (25.0%)		
5 ~ 10	137 (43.1%)	534 (38.2%)	285 (33.5%)		
≥10	66 (20.8%)	199 (14.2%)	102 (12.0%)		
Recruitment methods					
Established staff	68 (21.4%)	333 (23.8%)	234 (27.5%)	5.934	0.051
contract staff	250 (78.6%)	1,064 (76.2%)	618 (72.5%)		
Nurse safety behavior scores	45.81 ± 14.58	52.79 ± 4.68	58.99 ± 1.86	26.7	<0.001

Profile 1: low-calling group, Profile 2: medium-calling group, Profile 3: high-calling group.

### 4.5 Predictors of latent profile membership

A multinomial regression analysis was conducted in the study. The results showed that male nurses were less likely to belong to the “medium-calling “profile and the “high-calling “profile than compared to the “low-calling “profile (*OR* = 0.351, *p* = 0.011; *OR* = 0.284, *p* = 0.042), and nurses who did not have night shifts had a greater likelihood of belonging to the “medium-calling “profile (*OR* = 2.084, *p* = 0.007), the results are shown in Table 3.

**Table 3:** Multiple logistic regression analysis of different latent profiles of career calling in nurses (*n* = 2,567).

Variables	Profile 1 VS Profile 2			Profile 1 VS Profile 3		
	β	OR	95% CI	β	OR	95% CI
Gender						
Male	0.011	0.35	0.156–0.789	0.042	0.28	0.084–0.958
Female		−			−	
Age						
20 ~ 30	0.42	0.71	0.312–1.623	0.05	0.36	0.128–1.007
30 ~ 40	0.81	0.92	0.451–1.871	0.49	0.74	0.317–1.732
>40		−			−	
Professional title						
Nurse	0.19	0.36	0.077–1.660	0.20	0.33	0.061–1.819
Senior nurse	0.23	0.41	0.092–1.786	0.21	0.36	0.072–1.789
Supervisor nurse	0.31	0.47	0.111–2.021	0.25	0.40	0.085–1.903
Deputy/director nurse		−			−	
Number of night shifts per month						
None	0.007	2.08	1.227–3.539	0.06	1.92	0.964–3.816
1 ~ 5	0.2	1.33	0.856–2.065	0.37	1.33	0.713–2.462
5 ~ 10	0.88	1.03	0.701–1.514	0.79	0.93	0.529–1.62
≥10		−			−	
Years of work experience						
<5	0.93	0.97	0.468–1.994	0.58	1.32	0.494–3.519
5 ~ 10	0.82	0.93	0.51–1.707	0.89	0.95	0.434–2.066
10 ~ 15	0.54	0.84	0.485–1.46	0.66	0.86	0.43–1.706
≥15		−			−	

CI, Confidence intervals.

### 4.6 Comparing nurse safety behavior across career calling profiles

There were also significant differences in the latent profiles of career calling concerning their mean scores in nurse safety behavior (*p* < 0.001) (see Table 4). The mean scores of the nurse safety behavior in profiles 1, 2, and 3 were 3.818 (SD = 1.215), 4.399 (SD = 0. 390), and 4.916 (SD = 0.154), respectively. Moreover, the SNK test revealed that the mean score of the ‘low-calling’ group was significantly lower than that of the ‘medium-calling’ group and the ‘high-calling’ group, and the mean score of the ‘medium-calling’ group was significantly lower than that of the ‘high-calling’ group. A hierarchical regression analysis was conducted to explore the impact on nurse safety behavior. Nurse safety behavior was treated as the dependent variable, with a two-layer approach. In the first layer, control variables such as gender, age, highest degree, category of personnel, professional title, number of night shifts, and work experience are included. In the second layer, the latent categories of career calling were introduced. The first item of the independent variables was set to the reference group. The results revealed significant effects of different latent categories of career calling on nurse safety behavior. The results of hierarchical regression analysis indicated that gender, age, Professional title, Number of night shifts, and Work experience entered the regression equation model for nurse safety behavior (*F* = 3.519, R2 = 0.128, *p* < 0.001). Based on Model 1, distinct subgroups (profile 1 vs. profile 2: *β* = −0.461, *p* < 0.001; profile 1 vs. profile 3: *β* = 0.824, *p* < 0.001) were significantly associated with nurse safety behavior. Compared with those in profile 1, nurses in profiles 2 and 3 had a significantly higher nurse safety behavior score. The overall model was significant (*F* = 82.017, *p* < 0.001), and the subgroups of career calling explained an additional 30.7% of the variance in the nurse safety behavior (Δ*R*^2^ = 0.307), with the overall model explaining 31.0%. The result of the hierarchical linear regression analysis for the nurse safety behavior is shown in Table 5.

**Table 4:** Nurse safety behavior difference of three profiles of career calling.

	Nurse safety behavior (M ± SD)
Low career calling (profile 1)	3.818 ± 1.215
Medium career calling (profile 2)	4.399 ± 0.390
High career calling (profile 3)	4.916 ± 0.154
*Hc*	566.616
*P*	<0.001
*SNK*	1<2, 1<3, 2<3

M mean; SD, standard deviation; SNK Student–Newman–Keuls.

**Table 5:** Hierarchical linear regression analysis for the nurse safety behavior (*n* = 2,567).

Variables	Model 1					Model 2				
	*B*	*SE*	*β*	*t*	*P*	*B*	*SE*	*β*	*t*	*P*
Contant	4.398	0.099		44.514	0.000	3.818	0.086		44.374	0.000
Gender										
Female	0.072	0.090	0.016	0.797	0.425	−0.018	0.076	−0.004	−0.242	0.809
Age										
30 ~ 40	−0.002	0.041	−0.001	−0.045	0.964	−0.063	0.034	−0.050	−1.850	0.064
>40	0.070	0.065	0.042	1.080	0.280	−0.049	0.055	−0.029	−0.891	0.373
Professional title										
Senior nurse	−0.049	0.046	−0.038	−1.068	0.285	−0.026	0.038	−0.020	−0.669	0.503
Supervisor nurse	−0.057	0.050	−0.045	−1.141	0.254	−0.028	0.042	−0.022	−0.672	0.502
Deputy/director nurse	−0.105	0.080	−0.036	−1.316	0.188	−0.136	0.067	−0.046	−2.031	0.042
Number of night shifts/month										
1 ~ 5	−0.046	0.037	−0.032	−1.273	0.203	−0.018	0.031	−0.012	−0.573	0.567
5 ~ 10	−0.023	0.035	−0.018	−0.664	0.507	0.020	0.030	0.015	0.667	0.505
>10	−0.081	0.044	−0.045	−1.842	0.066	−0.007	0.037	−0.004	−0.202	0.840
Work experience										
5 ~ 10	0.049	0.048	0.034	1.018	0.309	0.059	0.040	0.042	1.480	0.139
10 ~ 15	0.122	0.055	0.093	2.216	0.027	0.108	0.046	0.082	2.340	0.019
>15	0.175	0.064	0.121	2.715	0.007	0.141	0.054	0.097	2.603	0.009
Career calling										
Medium career calling (profile 2)						0.580	0.033	0.461	17.755	0.000
High career calling (profile 3)						1.097	0.035	0.824	31.408	0.000
*F*			3.519**					82.017**		
*R* ^2^			0.128					0.557		
Adjusted R^2^			0.016					0.310		
ΔR^2^								0.307**		

^**^P < 0.001; SE, standard error; ΔR^2^, change in R^2^.

## 5 Discussion

This study was the first to clarify the career calling subgroups among Chinese nurses, investigate the factors associated with these subpopulations, and examine the relationship between nurses’ safety behaviors and the different career calling subgroups. This study offers significant advantages and practical implications. Firstly, this study highlighted the essential aspects of nurses’ career calling, uncovered latent subgroup structures, and identified the traits of different potential subgroups within nurses’ career calling. The findings contribute to the existing literature on the antecedents and theoretical frameworks related to career calling. In addition, we examined the individual and environmental factors that influence nurses’ sense of career calling. The findings enable nursing managers to tailor interventions for different subgroups of nurses based on their various levels of career calling, ultimately enhancing the level of career calling. Most significantly, the present study revealed that the profiles of career calling were positively associated with nurse safety behavior. This provides a unique perspective on optimizing nursing safety management strategies.

### 5.1 Status and characteristics of nurses’ career calling

In the present study, our results revealed that the career calling level among nurses was medium to high, which is similar to previous studies (Cao et al., 2019; El-Gazar et al., 2024). The possible reasons for this result may be related to the demography and work-related characteristics of the nurses in this research. The majority of nurses were senior nurses and supervisor nurses who had more than 5 years of working experience in this study. Younger nurses experienced lower feelings of calling (Pesonen et al., 2024). As career calling is a developmental construct, some studies have confirmed that nurses’ calling increases with years of work experience, and career calling is stronger among older nurses (Kallio et al., 2022). Along with the promotion of the professional title, nurses gained a stable career development and their career calling was raised (Praskova et al., 2014). According to SDT, autonomy, competence, and relatedness are the primary psychological needs of nurses in the workplace. Younger nurses may experience a diminished sense of belonging to work teams compared to the more experienced nurses, primarily because of their shorter tenure in the nursing team. Younger nurses limited work experience also leads to lower nursing competence, requiring them to rely on guidance from higher professional title nurses to carry out their tasks. This dependence can lead to feelings of restricted autonomy in their roles, ultimately diminishing younger nurses’ level of career calling. Conversely, experienced nurses and nurses with higher professional titles feel greater autonomy, competence, and relatedness, and thus they have higher levels of career calling. These results suggest that managers should be aware of the enhancement of career calling among younger nurses. And “Nursing is a calling” should be continually cultivated by hospital administrators throughout nurses’ professional careers (El-Gazar et al., 2024).

The results of this study indicated that a three-profile career calling model was appropriate. Based on the scoring responses, three profiles namely the “high-calling,” “medium-calling,” and “low-calling” emerged. Nurses in the high-calling profile got higher scores for item 2 (“I enjoy being a nurse more than anything else”), item 3 (“Being a nurse gives me immense personal satisfaction”), and item 12 (“Being a nurse is a deeply moving and gratifying experience for me”) compared to nurses in the low-calling profile. This result suggested that nurses who perceived high calling were more satisfied with their jobs, which is consistent with what previous studies have confirmed (Duffy et al., 2014; Li et al., 2021). Such results validated our research hypotheses that different profiles of career calling exist among nurses. Based on the COT, there are three types of causality orientations: autonomy orientation, control orientation, and impersonal or amotivated orientation. These findings suggest that nurses’ career calling is closely associated with the types of their causality orientations. Nurses in the high-calling profile may belong to the nurses with autonomy orientation, who act out of interest in and value what is occurring in nursing work. These nurses value personal career goals, self-determination, and autonomy, and are less likely to be influenced by external incentives. Thus, nurses in the high-calling tend to put in significant effort to achieve their nursing goals, seek greater autonomy, and enjoy a heightened sense of professional worth and fulfilment.

For nurses in the low-calling profile, the scores were all low, except for item 9 (“My current job is always in my mind in some way”). The score of item 4 (“I would sacrifice everything to be a nurse”) seems higher than other items. Based on the COT, nurses in the low-calling profile are more likely to have an impersonal or amotivated orientation, which is characterized by anxiety concerning competence. In complex nursing work scenarios, nurses in the low-calling profile are prone to perceive workload and stress. In the face of clinical nursing dilemmas, they tend to doubt their work abilities, thus displaying anxiety, and ultimately responding negatively. Because of the culture of self-sacrifice within the nursing profession, nurses may be more likely to experience job dissatisfaction, burnout, and retention problems, especially for younger nurses (Ciezar-Andersen and King-Shier, 2021). Moreover, nurses may worry about deviation or legacy of work with the work demands increasing, which reduces the level of the psychological detachment of nurses (Sagherian et al., 2023), leads to emotional exhaustion and turnover intention, and in turn decreases career calling (Wu et al., 2023).

It is worth noting for nursing managers that the majority of nurses in this study belong to the medium-calling profile. On the basis of the COT, nurses in the medium-calling profile may have control orientations or are somewhere in the middle between autonomy orientation and amotivated orientation. Therefore, there is room to improve these nurses’ career calling level. These results suggest that nursing administrators need to take a series of measures to meet nurses’ needs of autonomy, competence, and relatedness, thus helping nurses facilitate the internalization of external motivation into autonomous motivation, to promote the level of career calling among nurses.

In summary, the results of this study showed that there were distinct types and characteristics of nurses’ career calling, suggesting that nursing managers should adopt targeted interventions based on the characteristics of nurses to improve their career calling.

### 5.2 Factors influencing potential profiles of nurses’ career calling

Similar to previous studies, the results indicated that men were more likely than women to be in the low-calling profile (Jiang et al., 2023). The reasons may be related to the nursing profession’s gender segregation. The traditional model of nursing student training tends to be dominated by female students, which may not be conducive to the establishment of male students’ confidence in the nursing profession, leading to the loss of male nursing students (Twidwell et al., 2022). Previous studies have found that male nurses in the field faced comparatively greater professional responsibilities (Mao et al., 2021). Male nurses are more prone to feel depressed when their career development is being impeded, which lowers their career calling. Therefore, hospital managers should pay more attention to developing the professional identity of male nurses and measures should be taken to promote their career development.

Our study found that nurses who did not work night shifts tended to belong to the medium-calling profile compared to nurses who worked more than 10 night shifts per month. Nurses who participated in night shifts had a higher risk of low-level career calling compared to nurses who did not work night shifts. The reason may be that the workload and psychological pressure of nurses during night shifts are higher. Frequent night shifts can disrupt nurses’ biological clocks, impacting their sleep quality and family relationships (Kaplan et al., 2024). Conversely, nurses who do not have night shifts find it easier to relieve fatigue, and experience reduced psychological stress, hence enhancing their career calling. However, it is interesting to find that the effect of the number of night shifts did not seem to have a significant effect on nurses in the high-calling profile in this study. According to the COT, nurses with higher career calling tend to be driven by intrinsic motivation to undertake nursing work, they consider the nursing profession to be the meaning of their lives. Therefore, when faced with a job situation such as night shifts, nurses in the high-calling profile show more composure and are less vulnerable to negative environmental factors and remain motivated to work. Thus, nurses with a high level of career calling may be more inclined to utilize their strengths and address their deficits, resulting in a heightened sense of career success, thus weakening the negative impact of night shifts (El-Gazar et al., 2024). It suggests that the impact of night shift situations on nurses’ level of career calling needs to be further explored. And nursing managers should focus on the physical and mental well-being of nurses, strategically allocate human resources to enhance nurses’ efficiency and reinforce comprehensive skills training to boost nurses’ professional identity and elevate their career calling.

### 5.3 Latent profile differences in nurse safety behavior

Lastly, we found that career calling was an important predictor of nurse safety behavior and that it could independently explain 30.7% of the variation in nurse safety behavior after controlling for socio-demographic characteristics. The profiles of career calling were positively associated with nurse safety behavior; nurses in the moderate and high career calling groups had significantly higher levels of nurse safety behavior than nurses in the low-calling group. The higher the degree of career calling of the nurses, the higher the degree of nurse safety behavior. The results support our hypothesis 3 and are consistent with previous studies. Following the SDT, a strong sense of career calling serves as an important motivational factor that makes it easier for nurses to wholeheartedly embrace their roles and autonomously engage in patient care. Nurses with high levels of career calling exhibit a robust sense of professional identity, a stronger professional value orientation (Emerson, 2017), and consequently, a higher level of work engagement. In addition, nurses in high-calling profiles are more proactive in seeking support from colleagues and crafting their work to ensure the quality and safety of nursing care (Kallio et al., 2022). Thus, these nurses tend to perform better in safety behaviors. In contrast, nurses with low levels of career calling indicated lower levels of safety behaviors. This may be because nurses with low career calling tend to be less motivated to work. They are prone to be driven by impersonal or amotivated motivation. They believe that work is just a way to make money, which makes it hard to feel proud and inspired by their work (Zhang and Hirschi, 2021). Lack of work motivation can push nurses to adopt negative work attitudes and behaviors, such as burnout and lower safety behavior. Therefore, managers need to concentrate on those nurses with low levels of career calling and conduct professional education appropriately. It is important to improve nurses’ proper understanding of their profession and the sense of work value to promote safety behaviors.

In the present study, the career calling profile accounted for 30.7% of the variance in nurse safety behavior, and thus there may be additional predictors of nurse safety behavior. Firstly, prior research has shown that the workplace physical environment can impact nurses’ safety compliance and safety participation, improving nurses’ safety compliance in healthcare organizations (Al-Bsheish et al., 2023). Secondly, psychological factors may be an important predictor. A growing number of the literature suggests that psychological safety and psychological capital have a positive correlation with patient safety ratings (Cho et al., 2023; Elliott and Fry, 2021). There may be additional psychological factors (e.g., psychological resilience) that predict nurse safety behavior, which need to be further explored. Thirdly, prior studies have shown that organizational work factors such as teamwork (Han and Roh, 2020), patient safety climate (Seo and Lee, 2022; Moreno-Leal et al., 2024), and organizational learning culture (Abdallah et al., 2019) affect nurse safety behavior. The fourth is the supervisors’ leadership styles and behaviors. Research has confirmed that safety leadership (Subramaniam et al., 2023), transformational leadership (Boamah et al., 2018), inclusive leadership (Lee and Seo, 2024), and supervisor support (Seo and Lee, 2022) can impact nurse safety behavior notably. The above-mentioned factors were not considered in this study. Therefore, further research is necessary to investigate additional factors that predict nurse safety behaviors.

In sum, the result of this study implies that nursing managers should focus on the career calling level of nurses and implement suitable interventions based on nurses’ career calling profiles. For nurses with low levels of career calling, on the one hand, nursing managers should conduct professional education to enhance their understanding of the value of the nursing profession and improve their sense of professional identity, thereby stimulating intrinsic motivation to engage in nursing work. On the other hand, nursing administrators should prioritize the mental health of low-calling nurses by offering psychological support services to mitigate the negative impact of their emotional distress. For nurses in the medium-calling profile, nursing managers need to take an active leadership approach and develop a system of rewards and punishments to stimulate nurses’ intrinsic motivation and facilitate the internalization of external motivation into intrinsic motivation among nurses. For nurses with high career calling, nursing managers should further promote their professional success and career fulfilment by enhancing the workplace’s physical environment, improving the organizational work climate, and providing richer opportunities for career development. The above series of measures can improve nurses’ career calling, thereby promoting nurse safety behavior and ensuring patient safety in nursing care. Last but not least, nursing managers should organize strategies to optimize nursing safety management in accordance with the characteristics of different subgroups of nurses. For example, by enhancing safety training for young nurses to improve their safety behaviors (Yan et al., 2022). Nursing managers should also pay attention to the number of night shifts and the workload of nurses, optimize the nursing work environment through rational scheduling and providing more work resources, to stimulate intrinsic motivation and reduce the negative impact of workload on nurses’ safety behaviors.

## 6 Limitations

This study provides valuable insights into nurses’ career calling and its correlation with nurse’s safety behaviors. However, the study has certain weaknesses. Firstly, the cross-sectional design hindered the stability of the results from the analysis of potential profiles, thus longitudinal studies are needed to capture the evolutionary trajectory of nurses’ career calling in the future. Secondly, the reliance on self-reported data may lead to methodological biases and inaccuracy of responses, thus future studies should utilize larger and more diverse samples. Additionally, this study aimed to determine the relationship between career calling and safety behaviors among nurses without exploring all possible mechanisms, which highlights the importance of future research to reveal pathways and mechanisms between career calling and nurse safety behavior. Finally, the results of the study suggest that the level of nurses’ career calling was closely related to nurses’ psychological well-being and that a series of psychological counseling or psychological interventions may help to enhance the level of clinical nurses’ career calling. As this study did not involve the exploration of psychological interventions, future research needs to explore further the effectiveness of psychological measures to improve the level of nurses’ career calling to promote the sustainable development of the nursing profession.

## 7 Conclusion

In this study, the career calling of nurses was classified into three categories through latent profile analysis, namely, low-calling group, medium-calling group, and high-calling group; these three categories of career calling profiles have different influencing factors. By recognizing these categorical characteristics, hospital administrators can implement targeted management strategies based on the unique personality traits of nurses to enhance their career calling and clarify their career development. Additionally, this study has revealed that career calling is positively related to nurse safety behaviors. Policymakers and nursing managers should understand nurses’ characteristics of different career calling levels, provide more targeted guidance for appropriate understanding of work value, and develop interventions to facilitate work motivation to strengthen the quality of care.

## Data Availability

The original contributions presented in the study are included in the article/supplementary material, further inquiries can be directed to the corresponding author/s.

## Footnotes

^1^ www.wjx.cn
